# Assessment of Risk Factors Related to Environmental Factors and Herd Management for Bovine Respiratory Syncytial Virus and Bovine Parainfluenza Virus‐3 Infections Frequently Observed in Beef and Dairy Cattle

**DOI:** 10.1002/vms3.70299

**Published:** 2025-06-03

**Authors:** Ali Küçük, Yakup Yildirim, Bekir Çetintav

**Affiliations:** ^1^ Department of Virology, Faculty of Veterinary Medicine Burdur Mehmet Akif Ersoy University Burdur Turkey; ^2^ Department of Biostatistics, Faculty of Veterinary Medicine Mehmet Akif Ersoy University Burdur Turkey

**Keywords:** BPIV3, BRSV, Cattle, Risk Factors, RT‐PCR

## Abstract

**Background:**

Bovine Respiratory Disease Complex (BRDC) represents a multifactorial infection that poses a significant threat to animal health, leading to severe and fatal pneumonia outbreaks in large herds.

**Objective:**

In this study, the molecular diagnosis of Bovine Parainfluenza Virus Type 3 (BPIV3) and Bovine Respiratory Syncytial Virus (BRSV) was targeted in nasal swab samples collected from cattle with clinical signs of respiratory infections. Furthermore, this study aimed to identify possible environmental and herd management‐related risk factors associated with the occurrence of these infections in herds/farms where BPIV3 and BRSV were detected, using statistical methods.

**Methods:**

Therefore, a total of 200 nasal swab samples were randomly collected from 24 different cattle herds with respiratory infection outbreaks, representing 10% of each herd population.

**Results:**

In the study, which utilized the RT‐PCR technique, viral nucleic acid of BPIV3 was detected in 8% (16/200) of the samples, and BRSV was detected in 11.5% (23/200) of the samples. In addition, logistic regression models incorporating both fixed and multiple variables identified the following risk factors for BRSV infection: in the univariate regression analysis, quarantine status, air quality, the period of disease occurrence and previous occurrence of BRDC in the farm; and in the multivariate analysis, the disease period, herd size, bedding type and qualitative air quality were significant risk factors for the presence of infection in the sampled herds. For BPIV3, univariate regression analysis indicated that animal transport, housing type, ventilation and the duration from infection onset to sampling were risk factors, while in multivariate analysis, age, duration from infection onset to sampling, and air quality were identified as risk factors for infection in the sampled herds.

**Conclusion:**

A statistical relationship was demonstrated between BRSV and BPIV3 infections in cattle and certain herd, environmental and infection‐related factors, identifying these factors as risk factors contributing to the occurrence of these infections.

## Graphical abstract

In this study, molecular diagnosis of BRSV and BPIV3 factors was aimed and risk factors were tried to be determined by considering ‘environmental factors’, ‘herd management factors’ and ‘infection‐related factors.’

RT‐PCR technique was used for the molecular detection of pathogens and multivariate and univariate statistical analyses were performed using logistic regression and Chi‐squared tests.

According to the results of univariate analysis of BRSV prevalence, a statistically significant difference was observed between the two groups regarding quarantine status (*p* = 0.002), air quality (*p* = 0.009), season of disease occurrence (*p* = 0.039) and history of BRDC outbreaks in the herd (*p* = 0.033).

According to the results of multivariate analysis of BRSV statistically significant differences were observed in terms of season of outbreak occurrence, herd size, bedding type and air quality (qualitative).

According to the results of univariate analysis of BPIV3, statistically significant differences were observed between the two groups in terms of transport (*p* = 0.030), sheltering (*p* = 0.005), ventilation (*p* = 0.001) and duration of infection (*p* = 0.031).

According to the multivariate analysis of BPIV3, statistically significant differences were observed for age, presence of different animal species in the facility and air quality.

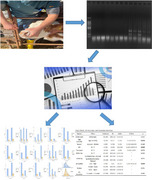

## 1 Introduction

Bovine Respiratory Disease Complex (BRDC) is a multifactorial infection that poses a serious threat to animal health in large herds through severe and fatal pneumonia outbreaks (Headley et al. 2018). Veterinary care costs, production losses, reductions in carcass weight and quality and high morbidity and mortality rates lead to substantial direct and indirect economic losses for producers (Irsik et al. 2006; Fulton 2009). Various abiotic factors such as transport, stress, environmental conditions (temperature fluctuations, poor ventilation, dust and harmful gases) and poor herd management, as well as host‐dependent factors like age, breed and immunosuppression, predispose animals to BRDC and complicate disease control efforts. The aetiology of this complex includes multiple viral and bacterial pathogens, such as bovine parainfluenza virus type 3 (BPIV3) and bovine respiratory syncytial virus (BRSV) (Küçük and Yıldırım 2022a).

BPIV3 is classified within the genus *Respirovirus* of the subfamily Orthoparamyxovirinae in the Paramyxoviridae family, with the causative agent named Bovine *Respirovirus* 3 (Rima et al. 2019). BPIV3 has nine proteins encoded by six genes. Of these proteins, N, M, P, F, HN and L proteins are structural proteins, while V, C and D are non‐structural proteins (Ellis 2010; Küçük and Yildirim 2022a). Fusion (F) and haemagglutinin–neuroaminidase (HN) are the most important surface proteins of BPIV. These proteins are responsible for the adsorption and penetration of the agent into the host cell (MacLachlan et al. 2017). The nucleocapsid (N) consists of the N protein, phosphoprotein (P) and large polymerase protein (L) that symmetrically wraps the viral genome. Another structural protein, the matrix (M) protein, is located between the nucleocapsid and the membrane. This protein plays an important role in the morphology of the agent and the stages of infection (Elankumaran 2013). Upon entering the body via inhalation, the pathogen initially replicates in the respiratory tract epithelial cells (Ellis 2010). Common symptoms include coughing, pneumonia, fever, anorexia, depression, nasoconjunctival discharge and abdominal breathing (Sharma and Adlakha 2009; Newcomer et al. 2017).

BRSV belongs to the genus *Orthopneumovirus* in the Pneumoviridae family with the renamed Bovine Orthopneumovirus. The virus, which has an enveloped and pleomorphic morphology, contains a negative‐sense single‐stranded (ss) unsegmented RNA genome of approximately 15.2 kb in length. The 10 open reading frames (ORFs) located on the viral genome code for 11 proteins (3′‐NS1‐NS2‐N‐P‐M1‐SH‐G‐F‐M2.1‐M2.2‐L‐5′). The membrane contains the large glycoprotein (G), the F protein and the small hydrophobic protein (SH). These proteins are responsible for the adsorption and penetration of the virus into the host cell (Ellis 2013; Küçük et al. 2024). In addition, there are genomic regions on the genome that encode the M protein, which forms a surface on the lower layer of the membrane and gives the virus its morphological feature, and the viral transcription termination factor M2‐1 (Valarcher and Taylor 2007). The virus primarily causes infections in autumn and winter (Rima et al. 2017). Transmission occurs through respiratory secretions (aerosols) from infected animals or via direct contact (Ohlson et al. 2010). The virus replicates primarily in respiratory epithelial cells, creating a range of infection presentations from subclinical to acute forms (Gershwin et al. 2008; Ellis 2009; Jia et al. 2021).

In this study, nasal swab samples were collected from cattle with signs of respiratory infection of different breeds, sex and ages to reveal the presence of BPIV3 and BRSV in the aetiology of respiratory system infections in cattle in the region where the study was conducted and the molecular diagnosis of BPIV3 and BRSV using the RT‐PCR method. In addition, data related to the farms, animals and infection periods during sampling were categorized under the main headings of ’herd‐related factors,’ ’environmental factors’ and ’infection‐related factors.’ Using logistic regression analysis, in which these data were designated as variables, this study aimed to obtain data on the conditions under which infections occur and identify potential risk factors influencing the occurrence of these infections. On the other hand, univariate analysis was performed using the independent *t*‐test for continuous variables and the Chi‐square test for categorical variables.

## 2 Materials and Methods

### 2.1 Animals and Sampling Used in Study

In this study, nasal swab samples were collected from 200 cattle across 24 different farms, representing 10% of each herd. These cattle with clinical signs of respiratory infection (e.g., dyspnoea, coughing, naso‐ocular discharge, temperature > 40°C) and had not been vaccinated against the targeted pathogens. The samples were transported to the laboratory under cold chain conditions in 1.5 mL PBS containing 2500 IU/mL penicillin and 20 mg/mL streptomycin. Of the sampled animals, 35.5% (71/200) were under 1 year of age, and 64.5% (129/200) were over 1 year. The distribution by sex and breed was 45.5% (91/200) female and 54.5% (109/200) male (57 calves and 52 steers), with 54% (108/200) being Simmental and 46% (92/200) Holstein.

### 2.2 Molecular Diagnosis

#### 2.2.1 Total RNA Extraction

Total RNA extraction from nasal swab samples was performed using the acid–guanidine–phenol method (Rio et al. 2010). The samples transported to the laboratory were thoroughly vortexed and then centrifuged at 2500–3000 rpm at +4°C for 20 min. After centrifugation, 250 µL of the supernatant was transferred into RNase/DNase‐free 2 mL microcentrifuge tubes, and 750 µL of Hibrizol (Hibrigen/Turkey) was added. The samples were stored at −80°C until the extraction phase. Total RNA extraction was carried out according to the manufacturer's instructions, after which the lyophilized RNA was rehydrated with 55–60 µL of DEPC‐treated water and stored at −80°C until the cDNA synthesis phase.

#### 2.2.2 Reverse Transcription‐PCR

Prior to PCR, viral cDNA was synthesized from viral RNA using the ABT One‐Step cDNA Synthesis Kit (ABT, Turkey, Cat No: C07‐01‐20), following the manufacturer's instructions. The G genomic region of BRSV and the M genomic region of BPIV3 were amplified using the RT‐PCR technique with gene‐specific oligonucleotide primers and PCR conditions as reported by Vilcek et al. (1994) and Küçük and Yildirim (2022b), respectively. Amplicons were observed using gel electrophoresis. The PCR amplicons of the samples were run on a 1.5% agarose gel at 90 V for 1 h and visualized under UV light using a transilluminator.

### 2.3 Potential Risk Factors

#### 2.3.1 Herd‐Related Potential Factors

Potential herd‐related factors were examined under five subheadings: ’Production type,’ ’Number of animals in the herd‘, ’Presence of different animal species on the farm‘, ’Quarantine practices’ and ‘Transportation status.’ Among the 24 sampled farms, the production types were 42% (10/24) dairy, 30% (7/24) beef and 29% (7/24) mixed. Herd sizes were distributed as follows: 50 head or fewer in 50% (12/24) of farms, 50–100 head in 17% (4/24) and over 100 head in 33% (8/24). Quarantine practices for sick animals or animals brought from other farms were not implemented in 75% (18/24) of the farms, while they were implemented in 25% (6/24). While no information was provided on animal movement in 42% (10/24) of the farms, movement was reported in 58% (14/24).

#### 2.3.2 Environment‐Related Potential Factors

Environmental factors were analysed under four subheadings: ‘Housing type’, ‘Bedding type’, ‘Ventilation type’ and ‘Air quality (qualitative).’ Of the sampled farms, 37% (9/24) had closed housing, while 62% (15/24) had semi‐open housing. Bedding types used included natural materials like straw or soil in 29% (7/24) and synthetic materials like concrete or pallets in 71% (17/24). Ventilation was natural in 58% (14/24) and artificial in 42% (10/24). Qualitative assessment of air quality (based on urine and faeces odour, etc.) indicated good quality in 37% (9/24) and poor quality in 62% (15/24) of farms.

#### 2.3.3 Infection‐Related Potential Factors

Infection‐related factors were classified under four subheadings: ‘Period of outbreak,’ ‘Number of animals showing clinical signs within the herd,’ ‘Time elapsed since the onset of clinical respiratory symptoms’ and ‘Previous occurrence of respiratory infections in the sampled herd.’ Respiratory infection symptoms were observed in 54% (13/24) of herds during autumn and in 46% (11/24) during the winter–spring period. The number of animals with clinical signs of respiratory infection was fewer than 10 in 67% (16/24) and more than 10 in 33% (8/24) of herds. In 67% (16/24) of herds, clinical signs appeared within 1 week, whereas in 33% (8/24), the duration exceeded 1 week.

### 2.4 Statistical Analyses

Statistical analyses were conducted using JAMOVI software version 2.3.2. Continuous variables were reported as mean ± standard error, while categorical variables were presented as counts or percentages. Univariate analysis was performed using the independent *t*‐test for continuous variables and the Chi‐square test for categorical variables. Mixed‐effect logistic regression models were applied to determine independent variables. Prediction models were developed in JAMOVI based on the identified risk factors, with the significance threshold set at *α* = 0.05.

## 3 Result

### 3.1 RT‐PCR Findings

**FIGURE 1:**
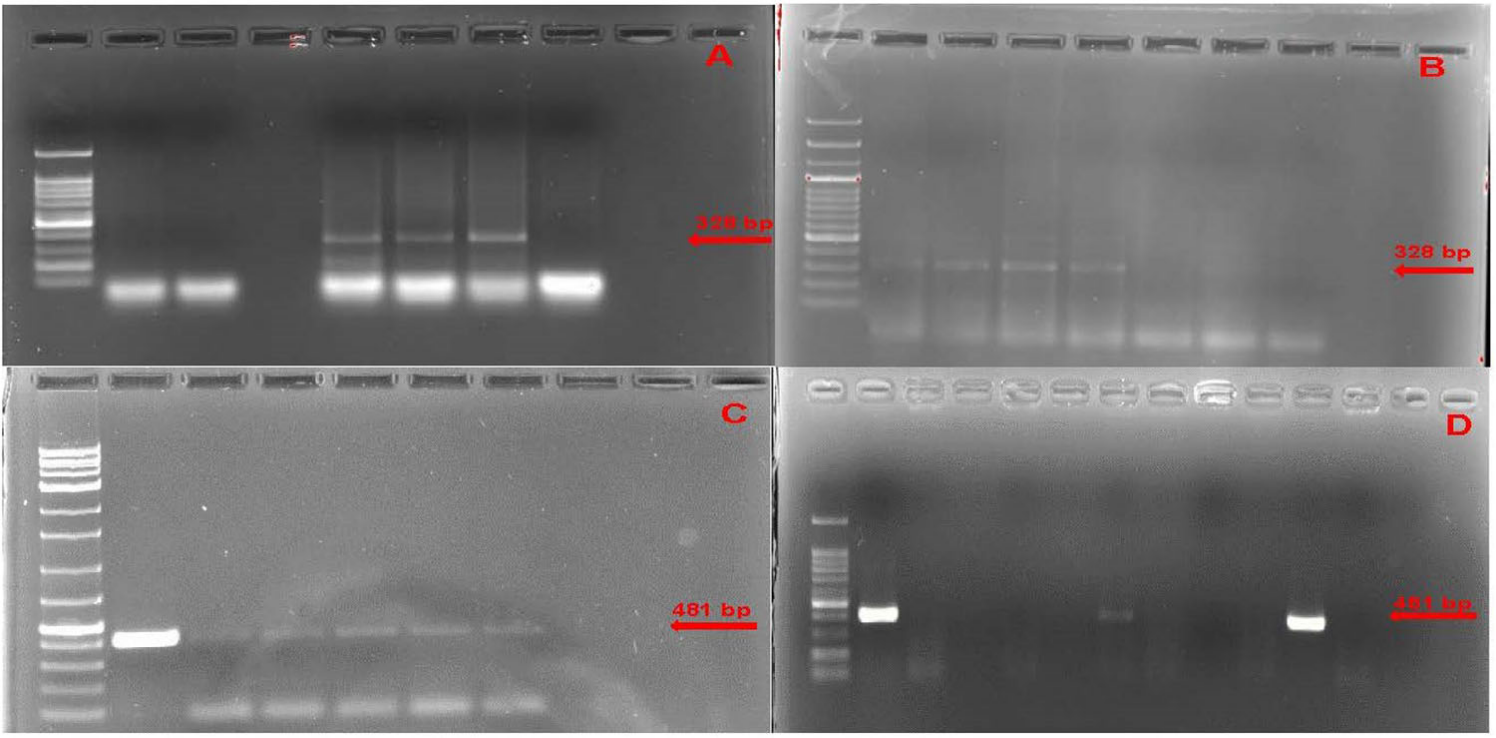
(A) BPIV3 gel electrophoresis image. (1): DNA ladder (100 bp), (2–3): negative samples, (4): negative control (5): positive control, (6–7): positive samples (328 bp), (B) BPIV3 gel electrophoresis image. (1): DNA ladder (100 bp), (2–4): positive samples (328 bp), (5): positive control, (6): negative control, (7–8): negative samples, (C) BRSV gel electrophoresis image. (1): DNA ladder (100 bp), (2): positive control, (3–7): positive samples (481 bp), (8) negative control, (9–10): negative samples, (D) BRSV gel electrophoresis image. (1): DNA ladder (100 bp), (2): positive control, (3): negative control, (4–6): negative samples, (7, 11): positive samples (481 bp), (8–10): negative samples.

Using the RT‐PCR technique in our molecular investigation, nucleic acid from BPIV3 was detected in 8% (16/200) and from BRSV in 11.5% (23/200) of the sampled animals (Figure 1). When evaluated at the farm level, BPIV3 was found in eight farms, and BRSV was found in nine farms. Both BRSV and BPIV3 nucleic acids were detected in the 8th, 13th and 14th herds where nasal swab samples were taken. In addition, both agents were found in the same nasal swab sample in two animals in the 8th and 13th herds. These farms were found to have substandard animal welfare conditions regarding care and feeding.

Among the farms where BPIV3 was detected, 75% (6/8) had enclosed housing, used synthetic bedding and had frequent animal movement without any quarantine measures for new or sick animals. Furthermore, 87% (7/8) had a noticeable odour of urine and faeces in the air. Respiratory infection symptoms in these farms appeared in 62% (5/8) during the autumn months, and infections in affected animals did not exceed 1 week at the onset.

For farms with BRSV detection, 67% (6/9) were semi‐open housing types and used synthetic bedding. Although there was animal movement in these farms, none implemented quarantine practices. In addition, 89% (8/9) of these farms had poor air quality, with respiratory outbreaks occurring in autumn. Information from farm owners indicated that 78% (7/9) of animals from which BRSV viral nucleic acid was detected were in the 1st week of infection.

### 3.2 Risk Factors Specific to BRSV

#### 3.2.1 BRSV Prevalence and Univariate Analysis

**FIGURE 2:**
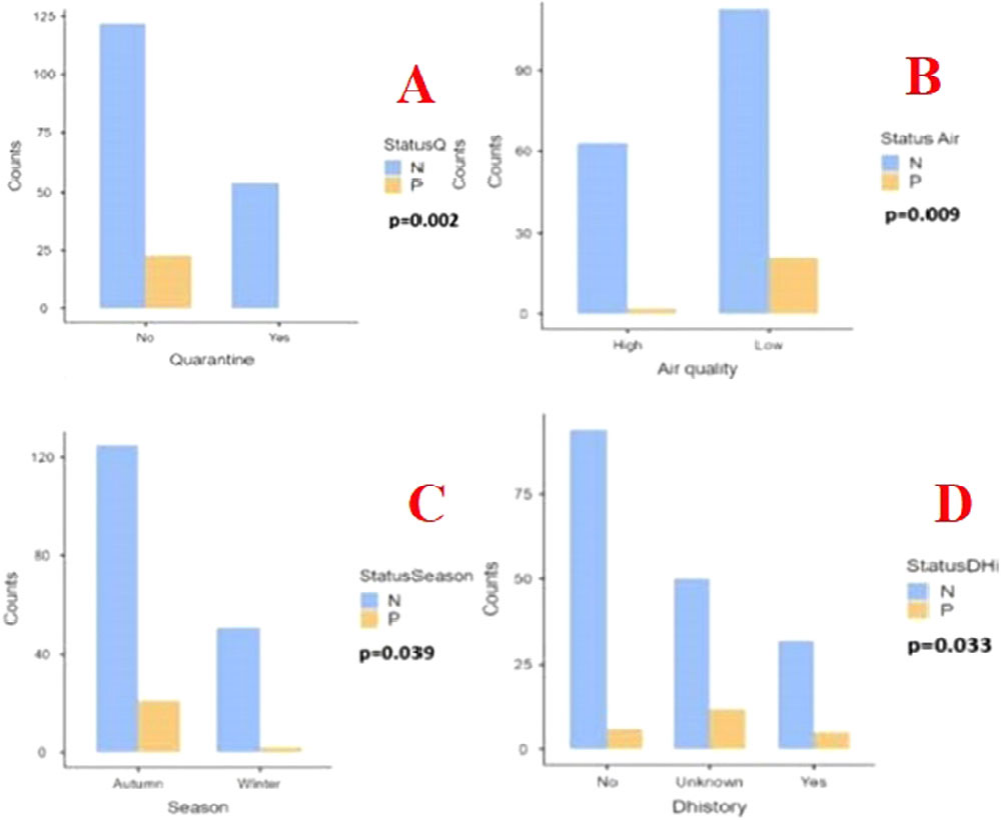
Graph showing the statistical relationship between the presence of BRSV in farms and quarantine practices in ranches Yes: Quarantine, No: Non‐Quarantine (A); Air quality in ranches High: Good air quality, Low: Poor air quality (B), season in which BRSV infection was observed (C) and previous BRDC outbreak in the ranches No: There has been no previous BRDC outbreak on ranches, Unknown: It is unknown whether there have been previous outbreaks of BRDC on ranches, Yes: There have been previous outbreaks of BRDC on ranches (D), N: BRSV Negative, P: BRSV Positive.

According to the results of univariate analysis, a statistically significant difference was observed between the two groups regarding quarantine status (*p* = 0.002), air quality (*p* = 0.009), season of disease occurrence (*p* = 0.039) and history of BRDC outbreaks in the herd (*p* = 0.033). The data is shown in Figure 2. These findings suggest that introducing animals purchased externally into the main herd without implementing quarantine measures could lead to the occurrence of BRSV outbreaks within the herd. The likelihood of detecting BRSV in herds increases when the air quality in animal shelters does not meet animal welfare standards. It is well‐established that the disease is predominantly observed during the winter and autumn months in the northern hemisphere. Consistent with this, the results of this study indicate a trend of increased detection of BRSV infections in herds during cold seasons. Respiratory system infections in cattle are often multifactorial, and the history of bacterial and viral infections shaped by farm conditions negatively impacts herd immunity, thereby contributing to the emergence of BRSV outbreaks.

#### 3.2.2 Multivariate Analysis of BRSV

In the model, independent variables included age, season of outbreak occurrence, presence of different animal species, transport status, herd size, bedding type, air quality (qualitative) and housing type. Age of sampled cattle was used as a covariate, while ranches were used as a clustering variable in the risk estimation model. According to the results of the multivariate analysis, statistically significant differences were observed in terms of season of outbreak occurrence, herd size, bedding type and air quality (qualitative) (Table 1).

**TABLE 1:** Fixed‐effects multivariate omnibus tests and parameter estimates for BRSV.

Names	Effect	Estimate	SE	Odds ratio	95% CI	*p*
Intercept	Intercept	−10.3623	66.9489	0.000	^a^	0.877
Age	Age	−0.0696	0.0386	0.933	0.865–1.006	0.072
Season of outbreak	Autumn–winter	4.7622	1.4309	117.006	7.083–1932.903	**<0.001** ^b^
Different kinds of animals	Y/N	−0.7427	0.8324	0.476	0.093–2.432	0.372
Transport	Y/N	−1.4442	1.2662	0.236	0.0197–2.822	0.254
Herd size	50–100; <50	−5.0665	1.7237	0.006	0.000–0.185	**0.011** ^c^
	>100; <50	−4.7317	1.7384	0.009	0.000–0.266	
Bedding type	Synthetic–natural	−1.9603	1.331	0.141	0.010–1.913	**0.033** ^d^
	Synthetic/concrete–natural/hay soil	0.3119	1.2589	1.366	0.116–16.108	
Air quality (qualitative)	Y/N	4.1116	1.4254	61.044	3.736–997.538	**0.004** ^e^
Sheltering type	Intensive–extensive	14.4097	200.8244	1.81E+06	^a^	0.227
	Semi‐extensive–extensive	15.9673	200.8234	8.60E+06	^a^	

Abbreviation: Y/N, yes/no.

^a^ Confidence interval could not be calculated when the odds value was close to 0.

^b^ The incidence of BRSV infections is higher in the autumn‐winter period than in other seasons.

^c^ The risk ofBRSV outbreaks is lower on farms with 100 or more animals than on farms withfewer animals.

^d^ Type of bedding has an effect, with synthetic/pallet bedding reducing the risk compared to natural/straw bedding.

^e^ Poor air quality increases the risk of BRSV infection in flocks.

The analysis identifies several significant predictors. The season of outbreak shows a substantial effect, with the autumn–winter period increasing the risk by 117 times compared to other seasons (OR: 117.006, 95% CI: 7.083–1932.903, *p* < 0.001). The number of animals in the enterprise is inversely associated with risk; farms with 50–100 animals (OR: 0.006, 95% CI: 0.000–0.185, *p* = 0.011) and over 100 animals (OR: 0.009, 95% CI: 0.000–0.266, *p* = 0.011) show significantly lower risks compared to smaller farms (<50 animals). It is thought that such a result is encountered because management can be more professional in large herds. Type of bedding also has an effect, with synthetic/pallet bedding reducing the risk compared to natural/straw bedding (OR: 0.141, 95% CI: 0.010–1.913, *p* = 0.033). Air quality is a critical factor, as low air quality increases the risk by 61 times compared to high air quality (OR: 61.044, 95% CI: 3.736–997.538, *p* = 0.004). These results revealed that BRSV infections can be seen in cold seasons when respiratory system infections are frequently seen, in situations where the number of animals in the herd tends to increase, and in barns where air quality and litter type are not suitable for animal welfare.

#### 3.2.3 BPIV3 Prevalence and Univariate Analysis

**FIGURE 3:**
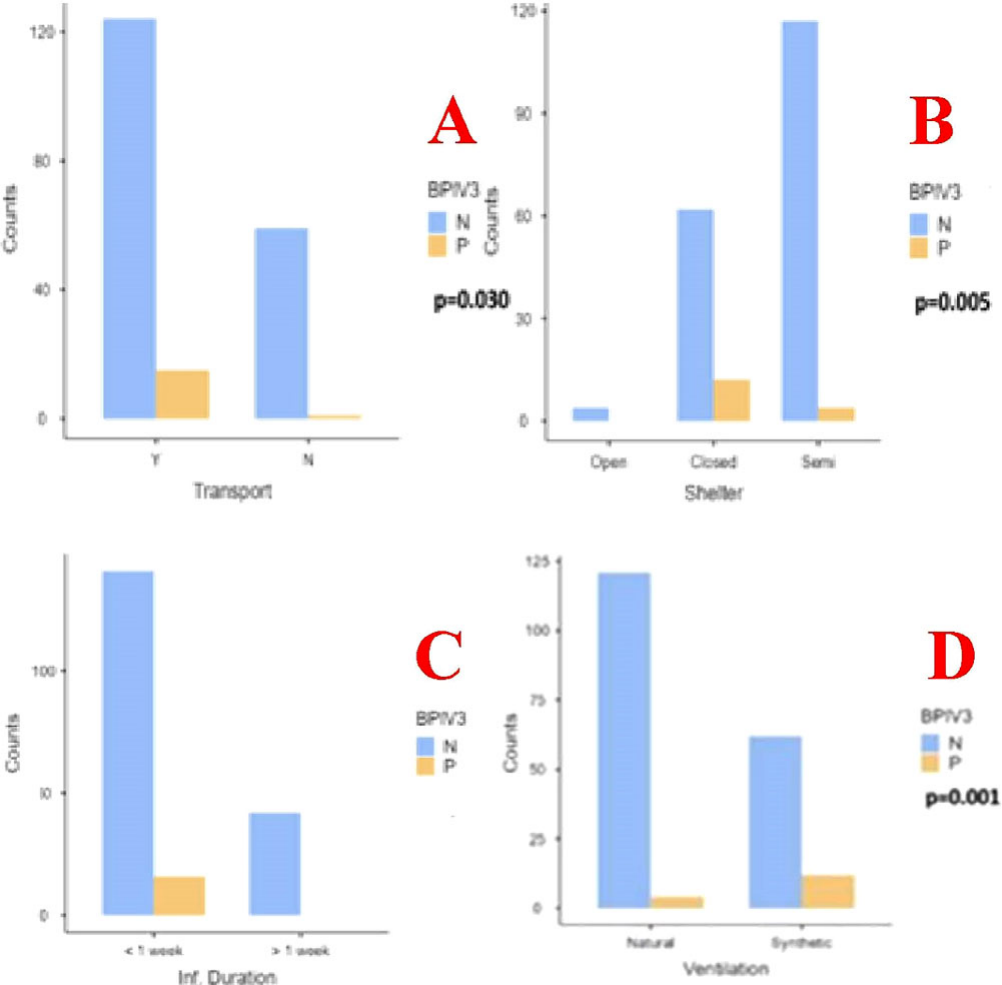
A graph display the statistical relationship between the presence of BPIV3 in herds and the variables transport history of animals, Yes: These animals transported, No: These animals were not transported (A), type of sheltering, Open: These animals are raised with free‐range feeding method, Semi: These animals are raised with semi free‐range feeding method, Closed: animals are raised in closed areas (B), type of ventilation in barn (C) and duration since the onset of the outbreak (D). N: BPIV3 Negative, P: BPIV3 Positive.

According to the results of univariate analysis, statistically significant differences were observed between the two groups in terms of transport (*p* = 0.030), sheltering (*p* = 0.005), ventilation (*p* = 0.001) and duration of infection (*p* = 0.031). The data is shown in Figure 3.

Research findings indicate that relocation and transportation, identified as one of the major immunosuppressive factors for cattle, increase the prevalence of BPIV3 within herds. Similarly, in cases of indoor (closed) housing with inadequate ventilation, BPIV3 prevalence shows a tendency to rise among cattle. Nasal swab samples collected during the 1st week of infection have been statistically proven to have a higher likelihood of detecting BPIV3 compared to samples obtained from cases exceeding 1 week of infection. This phenomenon is attributed to the high level of viral shedding during the initial week of infection, due to the local immune system not reaching sufficient titers during this period. Based on these findings, it may be advisable to isolate cattle with BPIV3 infection from the main herd during the first weeks of infection, as they are likely to shed high quantities of the virus.

#### 3.2.4 Multivariate Analysis of BPIV3

In the model, independent variables included age, season of outbreak occurrence, presence of different animal species, transport status, herd size, bedding type, air quality (qualitative) and housing type. Age was used as a covariate, while herd was used as a clustering variable in the risk estimation model. According to the multivariate analysis results, statistically significant differences were observed for age, presence of different animal species in the facility and air quality (Table 2).

**TABLE 2:** Fixed‐effects multivariate omnibus tests and parameter estimates for BPIV3.

Names	Effect	Estimate	SE	Odds ratio	95% CI	*p*
Intercept	Intercept	−46.465	244.526	6.61E‐21	^a^	0.849
Age (month)	Age (month)	−0.303	0.117	0.738	0.587–0.930	**0.010** ^b^
Season	Autumn–winter	1.655	1.766	5.232	0.164–166.839	0.349
Different kinds of animals	Y/N	4.756	2.306	116.249	1.266–10670.79	**0.039** ^c^
Transport	Y/N	−2.938	1.646	0.053	0.021–1.334	0.074
Underlay	Synthetic/hay–natural	−26.484	247.285	3.15E‐12	^a^	0.071
	Synthetic/concrete–natural	−20.444	247.286	1.32E0‐9	^a^	
Air quality	Low–high	4.396	2.02	81.157	1.547–4256.36	**0.030** ^d^
Shelter	Intensive–extensıve	42.784	323.495	3.81E+18	^a^	0.991
	Semi‐extensive–extensive	21.156	233.965	1.54E0+9	^a^	
Inf. duration	> 1 week; < 1 week	−27.452	378.637	1.20E‐12	^a^	0.942

Abbreviation: Y/N, Yes/No

^a^ Confidence interval could not be calculated when the odds value was close to 0.

^b^ The risk of BPIV3 infection decreases with increasing age.

^c^ The presence of different animal species on farms increases the risk of BPIV3 infection in herds.

^d^ The risk of BPIV3 infection is higher in barns with poor air quality than in barns with high air quality.

According to these results, it was determined that the probability of BPIV3 infection is higher in cattle under 1 year of age compared to cattle over 1 year of age. It is thought that this situation may be due to the inability to obtain sufficient colostral antibodies, especially in young people who are suckling, or the BPIV3 antibody titer in colostrum not being at a protective level. On the other hand, the fact that uniform animal breeding is not carried out in the enterprise and that the air quality in the shelters is so poor that it can cause irritation in the respiratory tract tissues can also be considered among the important immunosuppressive factors. Therefore, it was concluded that the prevalence of BPIV3 is higher in the enterprises mentioned above.

The results indicate that significant predictors in the model include Age (month), Presence of different animal types (Dif) and Air quality. Age has a protective effect, with each additional month reducing the risk by 26.2% (OR: 0.738, 95% CI: 0.587–0.930, *p* = 0.010). The presence of different animal types increases the risk significantly, with an odds ratio of 116.249 (95% CI: 1.266–10,670.79, *p* = 0.039), though the wide confidence interval suggests some uncertainty in this estimate. Poor air quality compared to high air quality increases the risk by approximately 81 times (OR: 81.157, 95% CI: 1.547–4256.36, *p* = 0.030). These findings highlight the critical role of age, animal diversity and air quality in influencing the risk.

## 4 Discussion

Respiratory infections in cattle, including pneumonia/bronchopneumonia‐related losses, contribute to direct and indirect economic losses for producers, especially in large herds. These include animal mortality, treatment and preventive costs, production loss due to reproductive issues and decreased carcass quality and weight. The aetiology of multifactorial respiratory infections in cattle commonly involves viral pathogens such as BRSV, BPIV3, BAV, BCoV, BVDV, BHV‐1 and IDV along with bacterial pathogens such as *Mannheimia haemolytica*, *Pasteurella multocida* and *Mycobacterium bovis* (Irsik et al. 2006; Fulton 2009). Environmental factors, stressors, age and the immune status of the host are all factors that can contribute to the outbreaks and complicate disease control efforts (Kurburic et al. 2014).

This study provides data on the prevalence of BPIV3 and BRSV in cattle with clinical signs of respiratory infections in Turkey's Western Mediterranean region, with findings indicating that the sampled animals were in the acute phase of infections. In our study, the RT‐PCR technique, a molecular detection method known for its higher specificity and sensitivity compared to antigenic and virological methods, was utilized (Küçük and Yıldırım 2022a).

Studies have reported the global prevalence of BPIV3 to range from 1.05% to 21.67% (Thonur et al. 2012; Çomaklı and Özdemir 2019; Pardon et al. 2020; Kamdi et al. 2020; Küçük and Yıldırım 2022b, Ozbek et al. 2024), while BRSV prevalence varies between 1.29% and 56.4% (O'Neill et al. 2014; Moore et al. 2015; Kishimoto et al. 2017; Timurkan et al. 2019; Ozbek et al. 2024). The findings of our study align with these values.

Although the presence of BPIV and BRSV has been demonstrated in Türkiye using different diagnostic methods, there is no national prevention or control programme for these infections (Yıldırım and Burgu 2005; Çomaklı and Özdemir 2019; Küçük and Yıldırım 2022b). This study will contribute to the identification of risk factors related to BRSV and BPIV3 infections and support the development of an effective control programme by revealing potential risk factors associated with these infections due to environmental, herd and pathogen‐related factors.

The multivariate omnibus logistic regression model used in our study identified the relationship between the presence of BRSV in herds and risk factors such as the outbreak season, herd size, bedding type (natural/synthetic) and air quality (qualitative‐good/poor). Notably, the autumn–winter season, during which outbreaks occurred, was found to have a significant impact on BRSV prevalence (OR = 117.006, 95% CI: 7.083–1932.903, Table 2). This finding is consistent with several studies (Hägglund et al. 2006; Luzzago et al. 2010; Pardon et al. 2020; Cusack 2023). Sudden temperature changes in the autumn and winter months can have an immunosuppressive effect, facilitating disease development. In addition, as cold temperatures, animals tend to cluster for warmth, which may enhance the transmission of pathogens through respiratory secretions and droplets. These factors are thought to contribute to the increase in outbreaks during the autumn–winter season. In our study, herd sizes were categorized as small (<50 cattle), medium (50–100 cattle) and large (>100 cattle), and a correlation was observed between increasing herd size and higher BRSV incidence (OR = 0.006, 95% CI: 0.000–0.185; 0.000–0.266, Table 2). These statistical findings are in line with research by Hussain et al. (2019), Ince et al. (2021) and Marutsov and Boneva‐Marutsova (2024). It is hypothesized that the larger herd sizes, especially in communal grazing cattle, contribute to outbreaks due to challenges in monitoring prophylactic measures, lack of control over animal sources and absence of pathogen testing in newly introduced animals. In addition, housing cattle beyond operational capacity may lead to immunosuppressive stress factors that further facilitate outbreaks. Our study demonstrated that bedding type, categorized as natural/synthetic and air quality defined qualitatively by factors such as ammonia odour, ease of breathing and the impact of ambient temperature on respiration are associated with the presence of BRSV in herds. Only one study has statistically evaluated these factors. Contrary to our findings, Pardon et al. (2020) indicated that air quality and bedding type do not constitute risk factors for BRSV outbreaks. Ammonia components resulting from urine accumulation are thought to have a corrosive effect on respiratory tract cells, potentially suppressing local immunity and facilitating pathogen replication in these areas. Bedding that impedes the removal of urine and faeces was also found to indirectly affect air quality. Particularly in farms using closed or semi‐enclosed feeding systems, this situation is believed to gradually deteriorate air quality, leading to immunosuppressive effects, such as respiratory distress and irritation of respiratory tissues, in animals over time.

In the fixed‐variable logistic regression model used in our study, a relationship was identified between the presence of BRSV in herds and factors such as quarantine practice for newly introduced animals, air quality within the shelter, the outbreak season and previous BRDC outbreaks in the herd. The season in which the outbreak occurred and the qualitative suitability of air quality were identified as potential risk factors in both logistic regression models. In our study, a prior history of BRDC in the herd was identified as a potential risk factor for BRSV. This finding aligns with studies reporting higher BRSV incidence in herds with a history of respiratory infections compared to those without such a history (respiratory diseases in early life are thought to increase the prevalence of BRSV infections). Possible explanations for these differences include variations in herd owner knowledge, care and feeding conditions, host immunity status, animal age and sample size. Uncontrolled animal movement is recognized as a risk factor for the spread of infectious diseases (Elvander 1996). Newly introduced animals, acquired through purchase, trade or other methods, are considered responsible for the transmission of BRSV. Previous studies have confirmed this reporting BRSV infections following the direct integration of new animals into the main herd without quarantine (Shirvani et al. 2012; Cusack 2023). Our findings are consistent with these studies. However, in a study by Ince et al. (2021), this factor was not identified as a risk. These differences in research outcomes are thought to arise from variations in the number of animals introduced and herd management practices. Further studies are needed to determine the impact of quarantine practices for newly introduced animals on BRSV infections.

According to the multivariate omnibus and univariate logistic regression models used in our study, a statistically significant difference was found between BPIV3 presence in the sampled herds and risk factors defined as outbreak season, animal age, air quality in animal shelters, transport status, housing conditions, ventilation type and the time elapsed since the onset of the outbreak. In a recent study where BPIV3 was virologically diagnosed using the direct immunofluorescence microscopy method and general risk factors were determined using the Chi‐square method, we found a statistically significant difference between the presence of BPIV3 infection in herds and factors such as the absence of quarantine for newly introduced animals and facilities not conducive to animal welfare (Küçük and Yıldırım 2024). In this current study, we aimed to achieve a more refined outcome by specifying and grouping the potential risk factors identified in our previous study. Similar results were obtained, showing consistency with our previous research. However, in the previous study, we did not detect a statistically significant difference between BPIV3 infection and variables such as breed, age or sex of the animals. In this study, however, infection frequency was found to be higher in animals under 1 year of age compared to those over 1 year (OR = 0.738, 95% CI: 0.587–0.930). León et al. (2019), in their serological study on combined cattle ranches in Colombia, investigated the seropositivity of BRSV, BoHV‐1, BPIV3 and BVDV and aimed to identify various risk factors associated with these infections. Their findings showed 98.6% seropositivity for BRSV and 47.1% for BPIV3. Using the Chi‐square technique, they reported a significant difference in BPIV3 seropositivity between female and 1‐2‐year‐old cattle. Pardon et al. (2020), in their study on the molecular detection of certain bacterial and viral agents from bronchoalveolar lavage samples, identified herd size and recent animal introductions into the herd as risk factors for BPIV3 infections. Hashemi et al. (2022) conducted a serological study in Iran focusing on risk factors at both herd and individual animal levels for BVDV, BPIV3 and BoHV‐1. Using a logistic regression model, they identified a statistically significant difference in BPIV3 seropositivity between autumn–winter and spring–summer seasons. However, due to the absence of a significant difference in samples from animals aged 1–4 years, they did not consider age as a risk factor.

In a large‐scale review conducted in Australia, Cusack (2023) demonstrated that the seropositivity of primary viral and bacterial agents of BRDC is higher in autumn–winter months compared to other seasons. In addition, feeding and sheltering conditions were identified as risk factors for respiratory infections. In our study, a statistically significant association was observed between the transportation status of animals and BPIV3 outbreaks in herds. Our findings are consistent with studies by Pardon et al. (2020) and Cusack (2023). Transport is considered one of the major sources of stress for cattle, potentially leading to an immunosuppressive state. Given BPIV3's subacute infection characteristics, infected animals may be introduced to the main herd without detection, allowing for the spread of infection (Spilki 2016; Küçük and Yıldırım 2024). Based on these findings, we believe there is a plausible association between BPIV3 infection and animal transport (Supporting information).

Sheltering type was also identified as a risk factor for BPIV3 infection. This finding is parallel with Cusack (2023), suggesting that sheltering type (intensive/semi‐intensive/extensive) could indirectly affect air quality and animal welfare, thus impacting pathogen transmission. Proper ventilation in barns is essential to prevent the spread of respiratory infections among animals. Artificial or natural ventilation systems aligned with zootechnical standards and animal welfare can help remove pathogens present as aerosols through air circulation. Therefore, the quality of ventilation in facilities is crucial for preventing BPIV3 outbreaks. The findings of this study align with our previous research, where BPIV3 diagnosis and associated risk factors were investigated using virological techniques (Küçük and Yıldırım 2024).

In our study, the time from infection onset to sampling day was defined as the “infection duration” and categorized as <1 week and >1 week. Research has shown that BPIV3 can be detected in respiratory secretions for 7–9 days (MacLachlan et al. 2017; Küçük and Yıldırım 2022b). During the 1st week, both local and general immune responses emerge, with detectable levels lasting 2–3 weeks (Sharma and Adlakha 2009). Later, humoural immunity (e.g., IgA and IgM) and cellular immune responses (e.g., macrophages and natural killer cells) eliminate the pathogen, reducing its detectability in nasal swabs. Thus, the 1st week of infection is crucial for transmission within the herd and is also the optimal period for sampling. In our study, the early phase of infection demonstrated a statistically significant association with both spread within the herd and detectability in nasal swab samples. The age factor was also identified as a significant risk factor in the multivariate BPIV3 model. Previous studies have reported that BPIV3 is more prevalent in younger animals compared to older ones (León et al. 2019; Hashemi et al. 2022; Cusack 2023). Our statistical data are consistent with these findings. Previous reports, methods used to determine respiratory infection risk factors, are primarily serological. Due to the challenges in detecting viral agents causing respiratory infections and their generally low prevalence, it is challenging to obtain specific statistical data. In addition, virological or molecular diagnostic methods require sampling during the acute infection phase for pathogen detection. The direct testing methods are neither economically nor practically feasible on a large scale, which explains the prevalence of serological methods in these studies. However, serological methods do not enable direct detection of the pathogen; hence, data obtained about current respiratory outbreaks in herds may be insufficient for identifying pathogen‐specific risk factors. We believe that our study, which utilized highly specific molecular techniques to detect pathogens directly in acutely infected cattle, provides a unique contribution to the literature, particularly in identifying pathogen‐specific risk factors.

## 5 Conclusion

By employing two different models for BRSV and BPIV3, we identified risk factors that significantly influence the occurrence of these diseases. Effective control of respiratory infections in herds necessitates strict management of animal movement into and out of facilities, along with rigorous implementation of quarantine measures. In addition, our findings indicate the importance of enhancing air quality through careful attention to bedding type and ventilation systems. For successful prevention and eradication of BRSV and BPIV3 infections, further studies focusing on herd‐ and facility‐specific risk factors are warranted.

## Author Contributions

**Ali Küçük**: writing – original draft, methodology, investigation, formal analysis, data curation. **Yakup Yıldırım**: methodology, investigation, data curation, methodology, investigation. **Bekir Çetintav**: statistics analysis, writing, validation.

## Ethics Statement

Approval was obtained from the animal testing local ethics committee of Burdur Mehmet Akif Ersoy University. The procedures used in this study adhere to the tenets of the Declaration of Helsinki (MAKU‐HADYEK 1098/26.04.2023)

## Consent

The authors have nothing to report.

## Conflicts of Interest

The authors declare no conflicts of interest.

### Peer Review

The peer review history for this article is available at https://publons.com/publon/10.1002/vms3.70299

## Supporting information



Supporting Information 1. Prevalence of BRSV and Univariate Analysis

Supporting Information

Supporting Information 2. Prevalence of BPIV3 and Univariate Analysis

Supporting Information

## Data Availability

All data are presented in manuscript itself. Further details may be obtained through mail alikucuk@mehmetakif.edu.tr or alikucuk13@gmail.com
