# Supplementary material for: Assessment of Risk Factors Related to Environmental Factors and Herd Management for Bovine Respiratory Syncytial Virus and Bovine Parainfluenza Virus‐3 Infections Frequently Observed in Beef and Dairy Cattle
Source: Vet Med Sci. 2025 Jun 3;11(4):e70299. doi: 10.1002/vms3.70299 (PMC12132865; doi:10.1002/vms3.70299)

Prevalence of BRSV and Univariate Analysis

Results of the analysis showed statistically significant differences in Quarantine Status (P=0.002), Air Quality (P=0.009), Season (P=0.039), and Disease history (P<0.001) between the 2 groups.

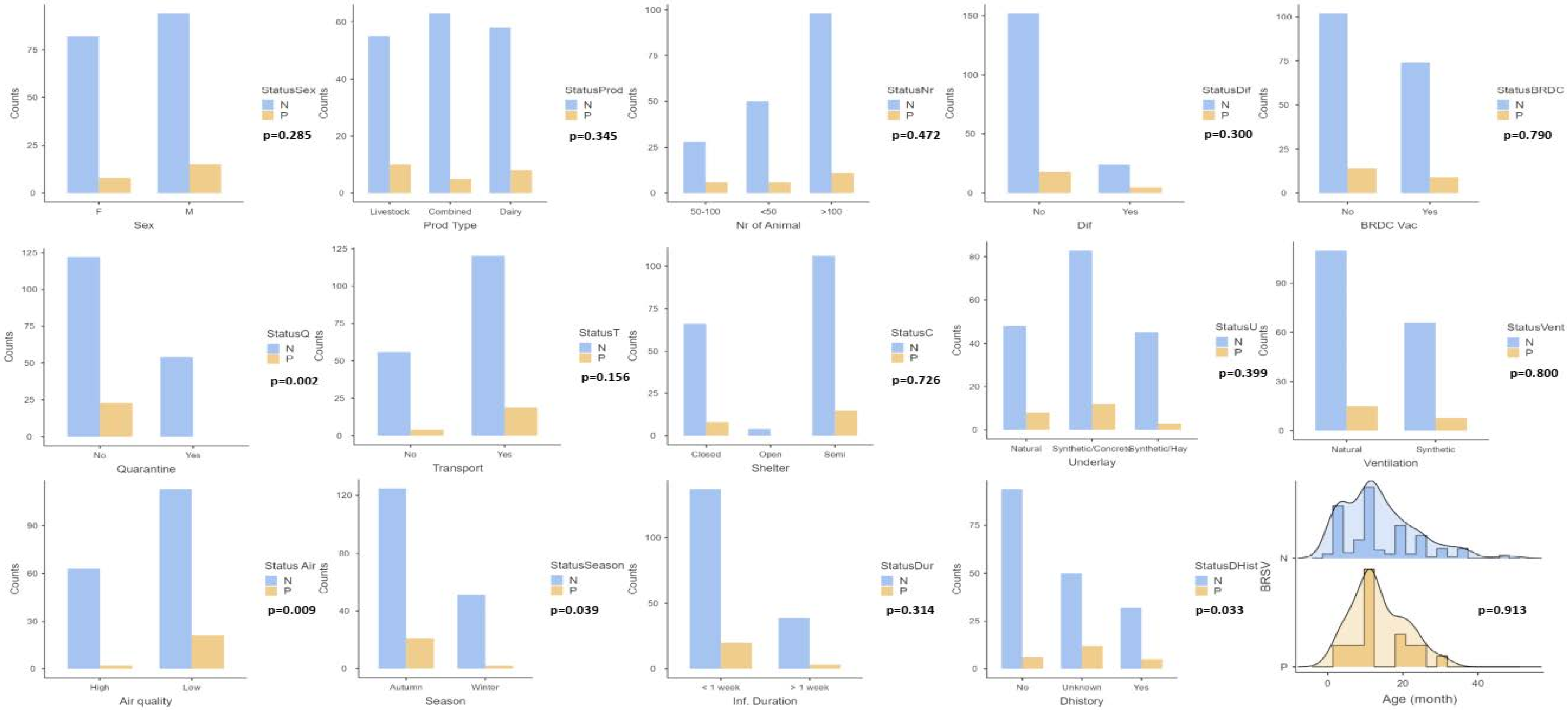

Supplement: Supplementary file 1 — Supporting Information 1. Prevalence of BRSV and Univariate Analysis [file VMS3-11-e70299-s001.pdf]
