# Supplementary figures and images for: Assessment of Risk Factors Related to Environmental Factors and Herd Management for Bovine Respiratory Syncytial Virus and Bovine Parainfluenza Virus‐3 Infections Frequently Observed in Beef and Dairy Cattle
Source: Vet Med Sci. 2025 Jun 3;11(4):e70299. doi: 10.1002/vms3.70299 (PMC12132865; doi:10.1002/vms3.70299)

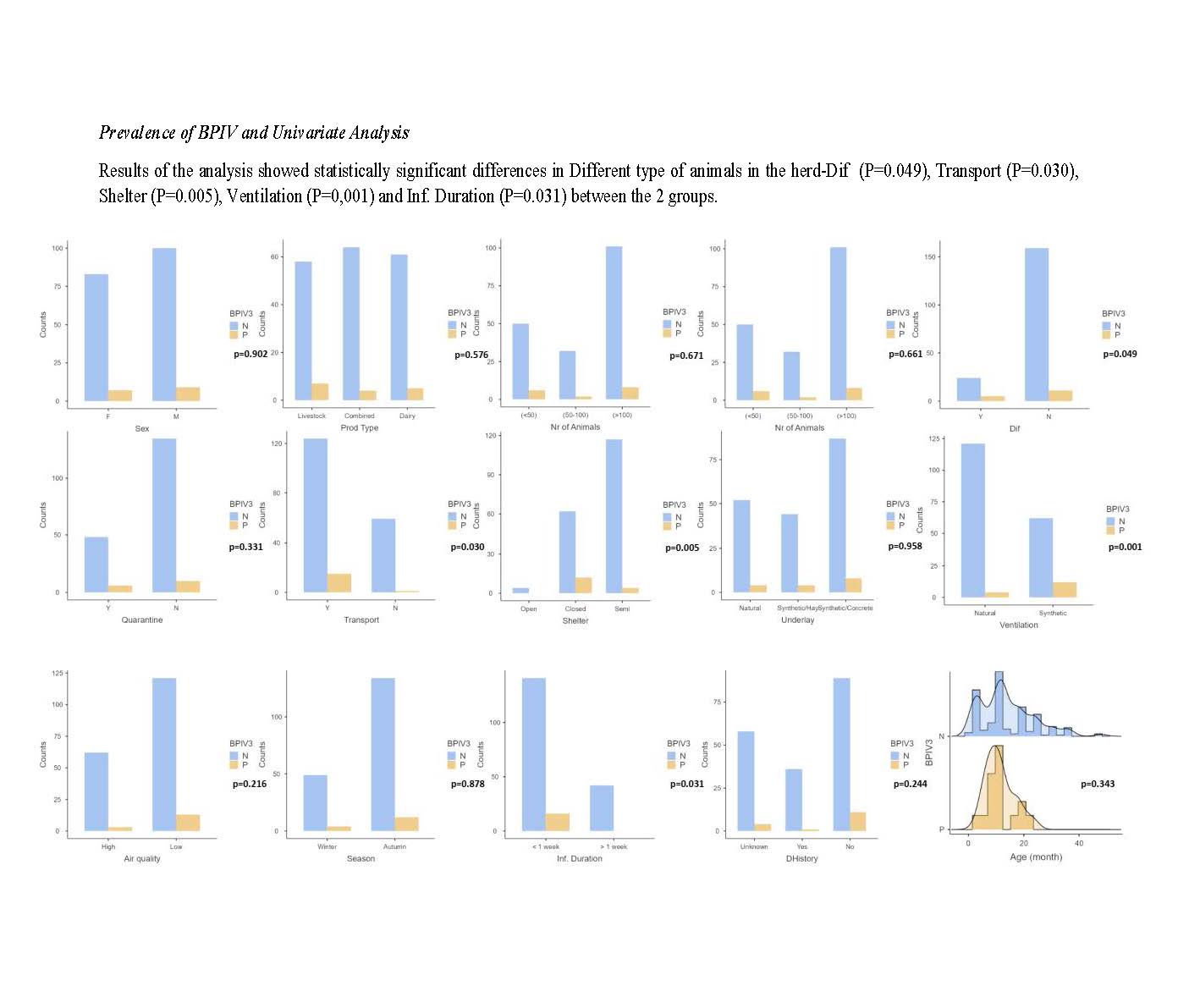

Supplement: Supplementary file 2 — Supporting Information [file VMS3-11-e70299-s002.jpg]

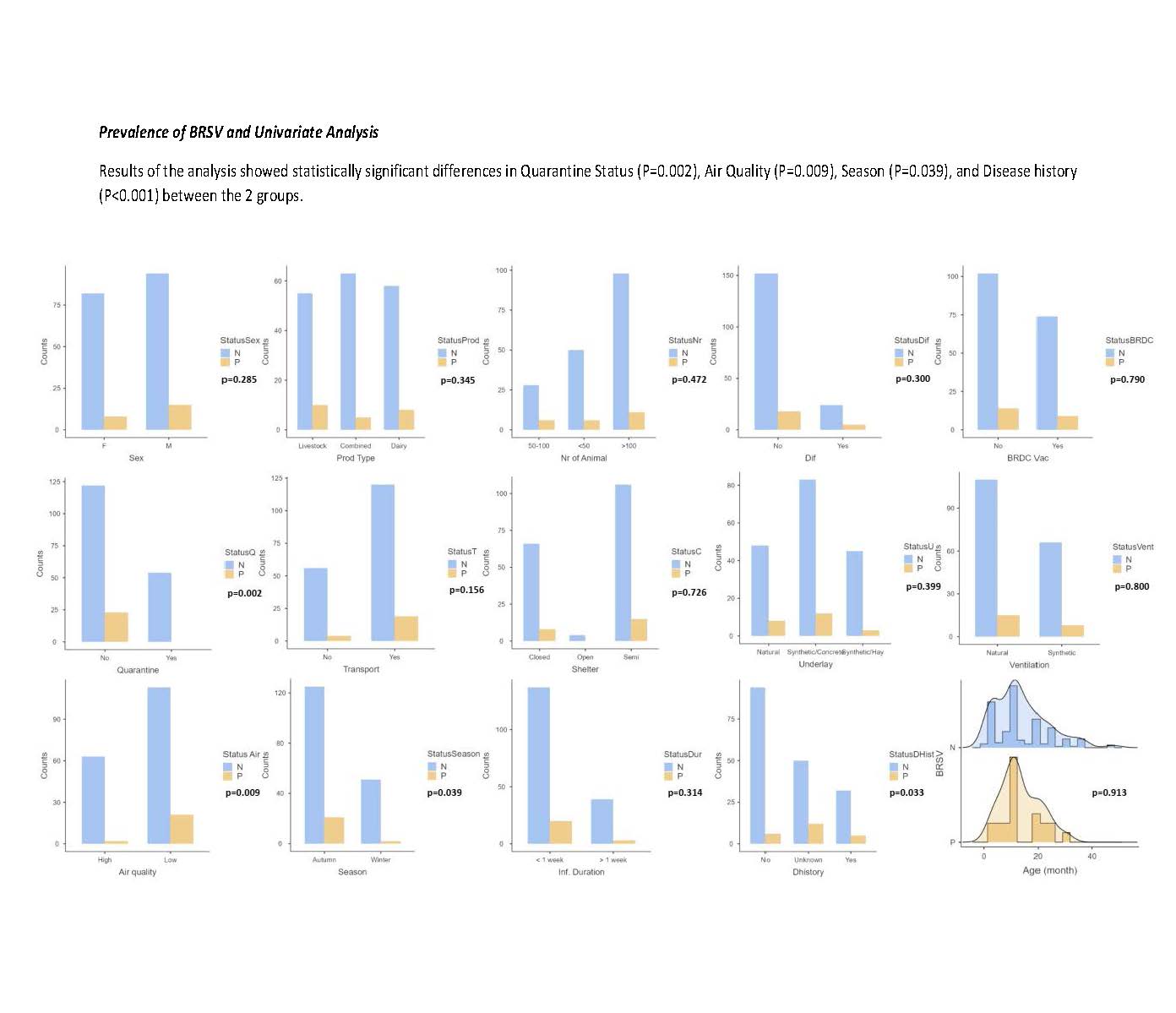

Supplement: Supplementary file 4 — Supporting Information [file VMS3-11-e70299-s003.jpg]
