# Supplementary material for: Assessment of Risk Factors Related to Environmental Factors and Herd Management for Bovine Respiratory Syncytial Virus and Bovine Parainfluenza Virus‐3 Infections Frequently Observed in Beef and Dairy Cattle
Source: Vet Med Sci. 2025 Jun 3;11(4):e70299. doi: 10.1002/vms3.70299 (PMC12132865; doi:10.1002/vms3.70299)

Prevalence of BPIV and Univariate Analysis

Results of the analysis showed statistically significant differences in Different type of animals in the herd-Dif (P=0.049), Transport (P=0.030), Shelter (P=0.005), Ventilation (P=0,001) and Inf. Duration (P=0.031) between the 2 groups.

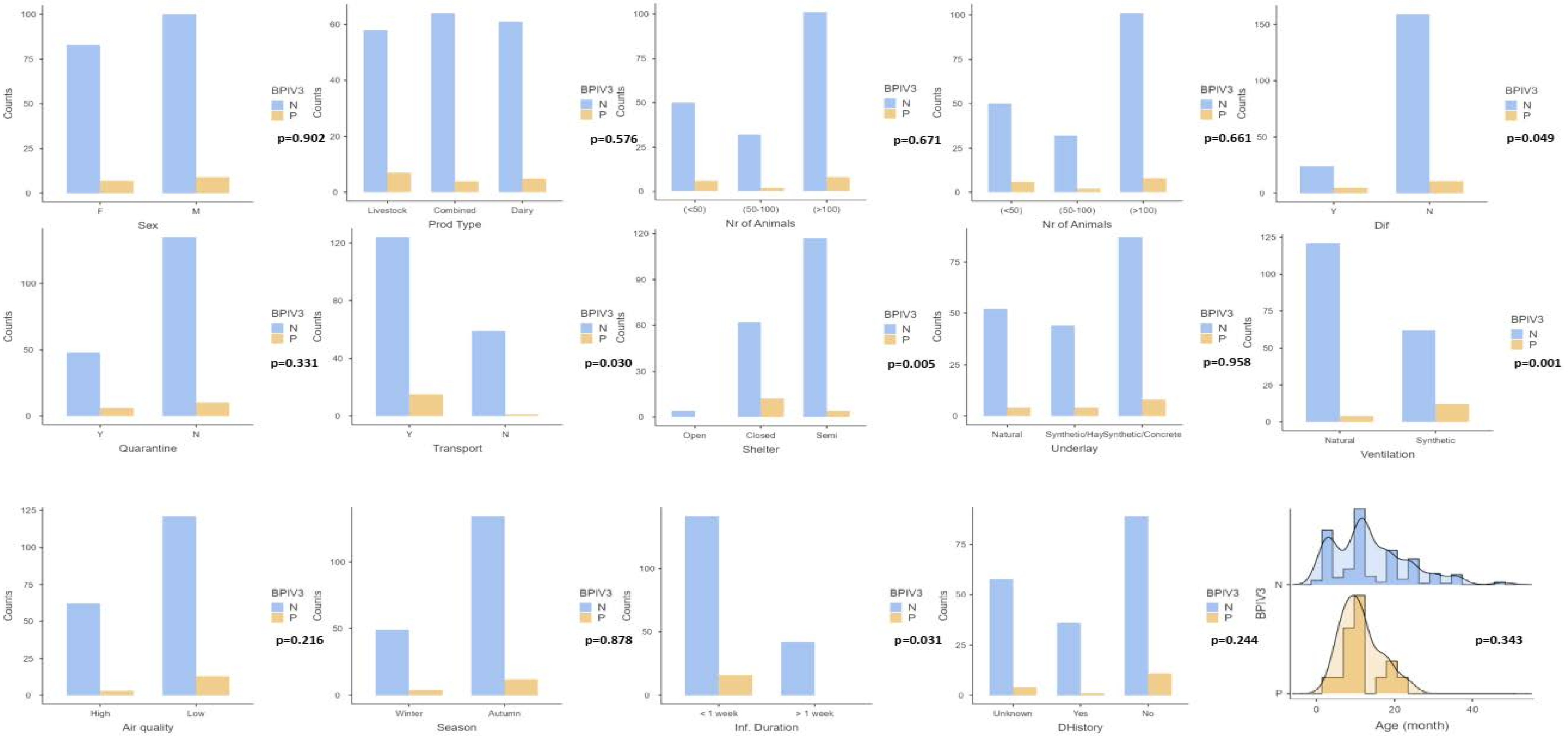

Supplement: Supplementary file 3 — Supporting Information 2. Prevalence of BPIV3 and Univariate Analysis [file VMS3-11-e70299-s004.pdf]
